# Supplementary material for: Clinical Outcomes, Patient-Reported Outcomes, and Economic Burden for Thai People Living with Chronic Urticaria (CORE-CU) in routine practice: A study protocol for a monocentric prospective longitudinal study
Source: PLoS One. 2023 Jan 20;18(1):e0279566. doi: 10.1371/journal.pone.0279566 (PMC9858793; doi:10.1371/journal.pone.0279566)
Supplement: Attachment — Submitted filename: 1.Response-to-Reviewer-PLoS-One-03-Dec-2022.docx [file pone.0279566.s002.docx]

**Response to Reviewers**

**PONE-D-21-27924R1 - [EMID:0dab44040bef7c5f]—entitled, “Clinical Outcomes, Patient-Reported Outcomes, and Economic Burden for Thai People Living with Chronic Urticaria (CORE-CU) in Routine Practice: A Study Protocol for a Monocentric Prospective Longitudinal Study”**

**Comments to the Author**

**Reviewer 1**

Thank you again for your work on the revised manuscript and the opportunity to review it. On the whole, you did a great job responding to the reviewer comments and I find the manuscript very much improved as a result.

Kindly thanks you for your valuable suggestion.

#1. I would like to offer one additional comment based on your revised text prompted by one of my original comments (#12). You have revised the sentence in question to read, "All data were anonymous and are not able to identify the participants' information." After reviewing this paragraph, I feel the key element to refine here has to do with the timing of the data use in question and subsequently the word tense to describe it.

"All data were anonymous" leads me to believe that this is an activity already taken and you were perhaps reviewing de-identified records as part of the screening process. But from the context, I believe you might be trying to convey thoughts about the security of the data you are assembling and using as part of this study. That is, you are maintaining the study data in a de-identified manner so any patient identifiers are not part of the analysis and the only way to know who a particular patient is would be to revisit a source document from a separate and secure location (locked file cabinet). If this is the case (referring to data security), the sentence could be made clearer. For example, "Data gathered are stored in the study database without identifiers and linking information is kept separate in a locked filing cabinet." Thank you again for your attentive response.

Thank you very much for your pertinent observation. We mage change the sentence per your recommendation accordingly under the “Informed consent, autonomy, and confidentiality” section as follows:

“**Data gathered are stored in the study database without identifiers, and linking information is kept separate in a locked filing cabinet. The unique identifiers will be generated, completely encrypted, coded, and will be recorded as double-entry for use, principally for statistical analysis purposes using computer software. Additionally, the collected data will remain confidential and shared only with authorized research members.**”

**Reviewer 2**

Thank you for addressing my previous comments. In reading the revision, I only have 3 remaining comments:

#1. Consider clarifying how participants' final status will be determined.

- There is a nice description of how reminders will be sent to participants at the time they are due for clinical follow-up. If the participant does not show up in clinic, how will the study team determine if they are deceased, transferred, or lost to follow-up?

- If none of these 3 dispositions are relevant to the given participant, will the follow-up be considered missed?

- When will the participant be considered a withdrawal? Is it if they actively state that they no longer wish to participate, or will the study team withdraw participants who continue to be no-shows to visits and/or unresponsive?

Thank you very much for your concerns. Ultimately, participants will be followed and censored until the date of the earliest incidence of death, withdrawal for any reason, transfer to another center, or loss to follow-up, whichever occurred first. Based on our routine follow-up care, the communications team and case manager staff members will contact patients/caregivers based on participants’ preference contact methods (text message, phone, social media, or direct mail). In the case where there is no response, participants will be closed out and censored from the study as a loss to follow-up.

Based on the Guideline for Good Clinical Practice, all participants have the right to withdraw informed consent at any time for any reason during the study period. In this case, data collected prior to study withdrawal or dropout due to any reason (based on the last observation carried forward) will be considered for participants who discontinue.

#2. When will analyses be performed? Will analyses be based on a subset of the database population, i.e., a study population? If so, how will inclusion in the study population be determined, e.g., based on complete data, etc.? The RESTORE guidelines suggest that a flow chart as a possible way to present this information.

Thank you very much for your comments. Preplanned statistical analysis will be performed based on one-year follow-up care. As mentioned, the last observation carried forward approach will be considered for data analysis. Regarding the whole sample population, multiple imputation methods will be used to analyze incomplete datasets for participants with missing covariates. However, variables containing more than 20% of missing data will be excluded from all analyses.

Moreover, for study reporting, we will disseminate the final results in line with the RECORD statement Guidelines (Item No.13)—an extension to the STROBE statement, which also includes the study flow diagram.

Reference

- Benchimol EI, et al; RECORD Working Committee. The REporting of studies Conducted using Observational Routinely-collected health Data (RECORD) statement. PLoS Med. 2015;12(10):e1001885. doi: 10.1371/journal.pmed.1001885.

#3. In the Informed Consent section, there are a few spots where the tense is confusing or mixed within sentence. You should consider revising this section to make it more clear that participants have already been consented and that the study team will continue to obtain informed consent, all per the ethical guidelines.

Thank you for your suggestions. To make it more clear, we have to rewrite the “Informed Consent” section as recommended.

Please read: “**Written informed consent will be obtained from all participants before they are included in the study. The participants’ consent will be obtained by trained research assistants. Based on the International Conference on Harmonization: Thai Guideline for Good Clinical Practice, a thumbprint is obligatory when a participant is illiterate. All participants have the right to leave a specific question unanswered or withdraw informed consent at any time for any reason during the study period.**”
